# P-1414. mRNA Vaccines for Tuberculosis Prevention: A Review of Current Research and Prospects

**DOI:** 10.1093/ofid/ofaf695.1601

**Published:** 2026-01-11

**Authors:** Tanzeela Sameen Saeed, Muhammad Ramish Saeed, Muhammad Fahad Abdullah, Muhammad Shoaib Qureshi, Armeen Saeed

**Affiliations:** Services Institute of Medical Sciences, Lahore, Pakistan, Fargo, ND; King Edward Medical University, Mayo Hospital Lahore, Lahore, Punjab, Pakistan; Allama Iqbal Medical College, Lahore, Pakistan, Lahore, Punjab, Pakistan; King Edward Medical University, Mayo Hospital Lahore, Lahore, Punjab, Pakistan; Allama Iqbal Medical College, Lahore, Pakistan, Lahore, Punjab, Pakistan

## Abstract

**Background:**

Tuberculosis (TB) remains a major global health burden, with 10.8 million cases and over 1.25 million deaths annually. The Bacillus Calmette–Guérin (BCG) vaccine provides limited protection against adult pulmonary TB, prompting the need for novel strategies. mRNA vaccines, successful in the fight against COVID-19, are now being explored for TB prevention.

**Methods:**

We conducted a comprehensive narrative review of the current state of mRNA vaccine development for TB. Literature was identified through structured searches in PubMed, Embase, the Cochrane Library, and ClinicalTrials.gov. Studies were included if they described immunological mechanisms, preclinical or clinical outcomes, vaccine delivery technologies, or challenges in TB-specific mRNA vaccine design. Emphasis was placed on both preclinical animal studies and early-phase clinical trials, as well as insights from major public health initiatives such as the WHO mRNA Technology Transfer Programme.

**Results:**

Preclinical studies demonstrate that mRNA vaccines elicit potent humoral and cell-mediated immune responses, surpassing BCG in efficacy in animal models. Key innovations include lipid nanoparticle (LNP) encapsulation, self-amplifying mRNA (saRNA) constructs, and epitope-specific designs that enhance immunogenicity. These platforms allow for rapid, scalable vaccine production and adaptability to emerging strains. The recent initiation of BioNTech’s Phase 1 clinical trial for BNT164, the first mRNA TB vaccine in human testing, represents a critical advancement in the field. However, challenges remain, including mRNA instability, cold-chain requirements, immune evasion by M. tuberculosis, and the limited translational fidelity of animal models to human disease.

**Conclusion:**

mRNA vaccines hold transformative potential for TB prevention, particularly in high-burden settings. Insights from the 2023 WHO mRNA Technology Transfer Programme meeting underscore the global prioritization of mRNA TB vaccines and the need for collaborative research, open data sharing, and equitable access. With continued innovation and investment, mRNA technology may become a cornerstone in global TB eradication efforts.

**Disclosures:**

All Authors: No reported disclosures

